# Assessment of potentially avoidable hospitalizations in Ningxia, China: application of a China-specific ACSCs list

**DOI:** 10.3389/fpubh.2026.1922200

**Published:** 2026-09-29

**Authors:** Jing Yang, Shiming Zhao, Nian Zheng, Yufan Yang

**Affiliations:** 1 Ningxia Hui Autonomous Region Health Commission Information Center, Yinchuan, Ningxia Autonomous Region, China; 2 Ningxia Medical University, Yinchuan, Ningxia Autonomous Region, China

**Keywords:** ambulatory care sensitive conditions, list application, potentially avoidable hospitalizations, primary health care, Western China

## Abstract

**Background:**

The China-specific list of ambulatory care sensitive conditions (ACSCs), comprising 14 conditions, represents the first evidence-based instrument for assessing primary health care (PHC) performance in China. However, this list has not yet been empirically applied to real-world data, and its feasibility and policy implications have not yet been evaluated.

**Objective:**

The aim of this study was to apply a China-specific list of ACSCs to systematically evaluate, for the first time, the scale, distribution, and determinants of potentially avoidable hospitalizations in Ningxia. This assessment aimed to provide a foundation for enhancing PHC performance and optimizing resource allocation in other regions of China.

**Methods:**

We extracted data from hospital discharge records in Ningxia for the period between 2022 and 2025. Generalized estimating equations were adopted to identify factors influencing potentially avoidable hospitalizations among patients.

**Results:**

The overall standardized rate of potentially avoidable hospitalizations in Ningxia was 505.37 per 100,000 population per year. Patients with chronic obstructive pulmonary disease (COPD) exhibited the highest rate of potentially avoidable hospitalizations (151.12 per 100,000 population per year). Local indicators of spatial association (LISA) identified Dawukou District and Jingyuan County as “high-high” hotspots, whereas Hongsibao District, Qingtongxia City, and Zhongning County were classified as “low-low” cold spots. Multivariate analysis indicated that age (65 years and older; OR = 4.367; 95% CI: 4.248–4.489), a high Charlson comorbidity index (CCI) score (3–4; OR = 4.557; 95% CI: 4.505–4.610), and the number of primary care beds per 1,000 population (OR = 1.166; 95% CI: 1.102–1.278) were significant risk factors for potentially avoidable hospitalizations. Conversely, female sex (OR = 0.786, 95% CI: 0.781–0.792), per capita gross domestic product (GDP) (OR = 0.846, 95% CI: 0.705–0.947), and the number of primary care physicians per 1,000 population (OR = 0.824, 95% CI: 0.742–0.915) were protective factors against potentially avoidable hospitalizations.

**Conclusion:**

Notable differences in the rates of potentially avoidable hospitalizations and disease burden were identified in Ningxia, accompanied by significant spatial clustering and variations among different populations. Strengthening the allocation of primary-care physicians is a key intervention for reducing potentially avoidable hospitalizations.

## 1 Introduction

Ambulatory care sensitive conditions (ACSCs) refer to health problems that can be effectively managed or even prevented through timely outpatient interventions, thereby lowering the likelihood of hospital admission (1–3). The hospitalization rate for ACSCs-related is widely utilized internationally as an outcome indicator for evaluating primary health care (PHC) and its quality (4–9). A lower hospitalization rate for ACSCs typically indicates more effective and accessible PHC services, whereas a higher rate suggests potential gaps in service or deficiencies in quality. Since the healthcare reform in 2009, China has made notable progress in expanding health insurance coverage and enhancing healthcare infrastructure (10); however, the capacity and effectiveness of PHC continue to encounter challenges, particularly in western and rural areas (11). These regions often experience a shortage of general practitioners, inadequate service capacity, low patient trust, and an imperfect tiered diagnosis and treatment system, all of which may contribute to an increased risk of potentially avoidable hospitalizations. It is worth noting that reducing the risk of potentially avoidable hospitalizations does not rely solely on single factors, such as primary health care capacity and physician density but rather depends on deeper structural mechanisms in service delivery, including the integrated allocation of health resources, care coordination across the social and healthcare sectors, and multidisciplinary team collaboration. Research by Giusti et al. indicates that this set of structural mechanisms is crucial for maintaining continuity of care, particularly for older adults aged 65 and older who have a heavy burden of comorbidity and are medically frail (12). To reduce potentially avoidable hospitalizations, it is necessary to do more than enhance the capacity of the healthcare sector alone; in particular, it is necessary to establish an integrated service network that bridges the boundaries between social and medical care. Against this backdrop, the development of a list of ACSCs tailored to China’s local context that can accurately identify potentially avoidable hospitalizations would have important practical policy implications.

Lists of ACSCs differ across various countries and research institutions. The initial definition and compilation of ACSCs were introduced by the Institute of Medicine (IOM) in the United States (U.S.) in 1993 (13). Since then, many organizations have published lists of ACSCs, including the U.S. Agency for Healthcare Research and Quality (AHRQ) (14), the Victorian Department of Human Services in Australia (15), the United Kingdom (UK)'s National Health Service (NHS) (16), the UK’s National Institute for Health and Care Excellence (NICE) (17), the Canadian Institute for Health Information (CIHI) (18), the Organization for Economic Cooperation and Development (OECD) (19), and the Australian Institute of Health and Welfare (AIHW) (20). These lists vary in terms of disease categories, inclusion criteria, and coding rules, reflecting the distinct disease profiles, health system characteristics, and data availability in different countries or regions. For instance, high-income countries typically include chronic diseases, whereas some low- and middle-income countries include vaccine-preventable diseases, such as measles and tuberculosis. This variability indicates that the ACSCs lists are not universally applicable and should be tailored to local contexts.

Despite the increasing recognition of ACSCs, China has historically lacked a validated list of diseases tailored to its specific context (21–23). In November 2025, Lai Hongyu, the Chinese ACSCs Working Group, Pan Jie, and their colleagues published the first evidence-based Delphi study, which reported a China-specific ACSCs list (24). This list comprises 14 diseases: five core diseases and nine general diseases (24). The list is methodologically rigorous, integrating a scoping review of 16 international ACSCs lists, a systematic review of more than 700 clinical guidelines, two rounds of modified Delphi surveys, and the Chinese Burden of Disease Assessment (24). However, since its publication, this list has not been assessed for application in any real-world population; thus, its evidence-based value, policy implications, and applicability across diverse populations remain untested.

The Ningxia Hui Autonomous Region, located in western China, presents a unique context for this study because of its limited resources. Within the region, significant geographic and socioeconomic disparities are evident: The northern Yellow River irrigation area has a relatively high level of economic development and relatively abundant medical resources, whereas the southern Xihaigu region experiences a shortage of medical resources and poor population health outcomes (25–27). These internal disparities enable an analysis of factors influencing potentially avoidable hospitalizations at the individual, service delivery, and systemic levels (28, 29). Additionally, Ningxia has developed a comprehensive regional health information platform that ensures complete coverage of hospital admission record data throughout the region, thereby providing a robust data foundation for assessing potentially avoidable hospitalizations using real-world data. The aim of this study was to utilize a China-specific list of ACSCs to conduct the first empirical assessment of potentially avoidable hospitalizations in Ningxia. It quantifies the rates of potentially avoidable hospitalizations, direct medical costs, and spatial distribution across various disease categories while also identifying relevant influencing factors. The objective of this study was to provide empirical evidence to promote the application of the ACSCs list in China and support enhancements in PHC and the targeted allocation of health resources in resource-constrained regions of western China.

## 2 Materials and methods

### 2.1 Study design and data sources

This study is a population-based retrospective observational analysis. Ningxia, located in western China, consists of five prefecture-level cities and 22 county-level administrative districts, with a population of approximately 7.3 million (25). The data utilized in this study included hospital discharge records from all healthcare institutions in Ningxia for the period from January 1, 2022, to December 31, 2025, as well as information from the Ningxia Health and Wellness Statistical Bulletin and the Ningxia Statistical Yearbook. Hospital admission records included demographic characteristics (age, sex, occupation, marital status, and type of health insurance). Notably, the New Rural Cooperative Medical Scheme and Urban Resident Basic Medical Insurance were merged in 2016 into the Urban and Rural Residents Basic Medical Insurance. The type of health insurance, such as the New Rural Cooperative Medical Scheme and Basic Medical Insurance for Urban Residents during the 2022–2025 period, is a legacy issue in the Inpatient Medical Record Cover Page System, which still follows the 2011 national standard. We recoded all types of health insurance, merging the original codes “Urban Resident Basic Medical Insurance” and “New Rural Cooperative Medical Scheme” into “Urban and Rural Residents Basic Medical Insurance.” Two researchers independently cross-checked the recoding process, conducting a secondary review of medical insurance records containing anomalies, omissions, or logical inconsistencies. They cross-referenced these records against the guidelines for completing the first page of medical records to validate the recoded data, minimizing the risk of classification errors that could result from the coding conversion. Clinical information [primary diagnosis, secondary diagnoses, admission and discharge dates, surgical procedures, Charlson Comorbidity Index [CCI] (30, 31)] and financial information (total costs) were collected. Data concerning regional PHC resources (number of primary care physicians per 1,000 population and number of primary care beds per 1,000 population), economic level (regional gross domestic product [GDP] per capita), and population size were obtained from the Ningxia Health and Wellness Statistical Bulletin and the Ningxia Statistical Yearbook. The Charlson Comorbidity Index (CCI) is calculated based on secondary diagnoses. The primary diagnosis used to determine ACSCs outcomes for each hospital admission record is excluded from the CCI calculation; all other secondary diagnoses (regardless of whether they appear on the ACSCs list) have been retained because they represent the patient’s actual burden of comorbidity (e.g., a patient hospitalized for COPD who also has diabetes). Excluding this information would lead to an erroneous underestimation of the patient’s baseline risk and fail to fully reflect the patient’s underlying comorbidity burden. Direct medical costs were defined as the “total cost” recorded in hospital discharge records, expressed in 2025 RMB, and adjusted according to the Ningxia Medical Consumer Price Index. Patients did not participate in the development of outcome measures during the study, and all analyses excluded any information that could identify individual patients. This study received approval from the Ethics Review Committee of the Information Center of the Health Commission of the Ningxia Hui Autonomous Region. Given that the study employed retrospective, deidentified administrative data, the need for informed consent was waived.

### 2.2 Study population and outcomes

The study population included all hospitalized patients. The primary outcome measure was the rate of potentially avoidable hospitalizations, which this study used as a population-level indicator to assess the burden of potentially avoidable hospitalizations in the region. The value of this indicator lies in identifying opportunities for systemic improvement at the macro level rather than providing a clinical determination of whether any specific individual hospitalization was avoidable. The criteria for this assessment are based on the list of 14 outpatient-sensitive conditions specified in the framework established by the Chinese ACSCs Working Group (24). Senior medical records coders and clinical experts were invited to review the list of ICD-10 codes corresponding to the 14 ACSCs conditions to confirm the completeness and accuracy of the code coverage. ICD-10 code mapping (Table 1) was used to classify outcomes; outcome classification relied solely on the primary diagnosis field and did not include the secondary diagnosis field. Using SPSS software, the ICD codes for the primary diagnosis in the medical records database were automatically matched against the ACSCs code list. If the ICD-10 code for the primary diagnosis fell within the ICD code range of any of the 14 ACSCs conditions, the hospitalization was classified as potentially avoidable hospitalizations (Outcome = 1); if the ICD-10 code for the primary diagnosis was not within the ACSCs code range, the hospitalization was classified as a non-ACSCs-related hospitalization (Outcome = 0). Records with missing, truncated, or invalid ICD-10 codes for the primary diagnosis were excluded from the outcome determination. Additionally, outpatients and emergency department patients, as well as any inpatients with ICD diagnostic codes related to pregnancy, labor or delivery, the postpartum period, the neonatal period, or newborns, were excluded. The rate of potentially avoidable hospitalizations was calculated by dividing the number of ACSCs patients (numerator) by the permanent resident population and then multiplying by 100,000. Using the 2020 Ningxia Population Census as the reference population, sex- and age-standardized potentially avoidable hospitalization rates were computed using the direct standardization method (32–36).

**Table 1:** China-specific ambulatory care sensitive conditions list and ICD-10 code mapping.

Disease	Category	ICD-10 code range
Chronic obstructive pulmonary disease (COPD)	Core	J40–J44
Bronchial asthma	Core	J45–J46
Hypertension	Core	I10, I11.9
Chronic kidney disease	Core	N18, I12, N03, Z49.1
Diabetes	Core	E10.0–E10.9, E11.0–E11.9, E12.0–E12.9, E13.0–E13.9, E14.0–E14.9
Bronchiectasis	General	J47
Chronic heart failure	General	I11.0, I13.0, I13.2, I50
Atrial fibrillation	General	I47.1, I47.9, I48, I49.5, I49.8, I49.9, R00.0, R00.2, R00.8
Chronic hepatitis B	General	B16.1, B16.9, B18.0, B18.1
Tuberculosis	General	A15, A16
Iron-deficiency anemia	General	D50
Primary osteoporosis	General	M80, M81
Gastroenteritis	General	A01–A04, A08, A09, E86, E87.6, K30, K52.2, K52.8, K52.9
Influenza	General	J10, J11

Following the consensus framework of the China ACSCs Working Group (23).

### 2.3 Statistical analysis

All the statistical analyses were conducted using IBM SPSS Statistics 27.0, Stata 17.0 and Geoda software. Two-tailed tests were employed, with a significance level established at *α* = 0.05. Chi-square tests were used to assess significant differences in categorical variables between the potentially avoidable and nonavoidable hospitalization groups. Spatial autocorrelation analysis was executed using Geoda software, and local Moran’s indices, along with their statistical values, were computed.

To avoid instability in the estimation of the random-effects variance components and severe bias in the standard errors of the macrolevel independent variables that can occur when a two-level mixed-effects model with a small number of clusters at the county-level (*n* = 22) is used, a binary logistic regression model based on generalized estimating equations (GEE) was used for analysis. This model estimated the effects of patient-level and county-level variables on potentially avoidable hospitalizations. The database of inpatients’ medical records that was used in this study contains de-identified unique patient identifiers that allow for the identification of multiple inpatient records related to the same patient over the 4-year study period. All of the eligible hospitalization records were included in the analysis; duplicate hospitalizations were not excluded. Given the internal correlation among multiple hospitalization observations for the same patient, a GEE model was adopted for regression analysis; the patient’s unique identifier was used as the clustering variable, an exchangeable working correlation matrix was specified, and robust sandwich variance estimates were employed to obtain robust standard errors. This approach addressed the issue of within-patient correlation in multiple hospitalization observations for the same patient.

The dependent variable was whether a patient experienced potentially avoidable hospitalization. Patient-level variables included age (categorical variable, with 0–15 years as the reference group), sex (categorical variable, with males as the reference group), marital status (categorical variable, with unmarried as the reference group), occupation (categorical variable, with employees of government agencies, enterprises, and public institutions as the reference group), types of health insurance (categorical variable, with Urban Employee Basic Medical Insurance (UEBMI) as the reference group), and the CCI (categorical variable, with a score of 0 as the reference). County-level variables included the number of primary care physicians per 1,000 population (continuous variable), the number of primary care beds per 1,000 population (continuous variable), and per capita GDP (continuous variable).

Because this study included only 22 independent counties and districts—a relatively small number of clusters—relying solely on robust variance estimation based on patient clusters carries the risk of downward bias in the standard errors of county-level covariates and excessively narrow confidence intervals. To address this issue of inference in small clusters, we used Stata 17.0 to conduct a wild cluster bootstrap analysis, with county and district IDs serving as the cluster units. The bootstrap resampling was performed at the county/district level without altering the point estimates of the regression coefficients in the GEE model; it only corrected the standard errors, 95% confidence intervals, and *p*-values for county-level macro variables. Ultimately, statistical inferences for county-level variables were based on the results, corrected by the wild cluster bootstrap method, while results for individual-level variables were based on the GEE analysis.

### 2.4 Construction of the spatial weight matrix

To characterize the spatial relationships among districts and counties, this paper constructs a first-order Queen adjacency weight matrix. Under this rule, districts and counties are considered adjacent if they share a common boundary or have an intersection. Compared to the Rook criterion, which determines adjacency solely based on shared boundaries, the Queen adjacency rule incorporates a wider range of spatial relationships, thereby more fully reflecting the spatial interaction effects between the regions. After the initial weight matrix was obtained, row normalization was performed to ensure that the sum of weights in each equaled 1. This eliminated biases caused by the uneven number of neighbors among study units and ensures the horizontal comparability of spatial autocorrelation results. The LISA index (37) was selected for the local spatial autocorrelation analysis, with a significance threshold set at 0.05. Significance results were obtained using 999 Monte Carlo resampling tests.

It should be noted that the spatial analysis in this study was based on inpatient medical record data from 22 counties and districts within the Ningxia region and does not include information on patients seeking medical care across provincial borders. Residents of certain counties who sought medical care across provincial borders were not included in the calculations of the county-level rates of potentially avoidable hospitalization in this study, which may have resulted in an underestimation of the actual amount of potentially avoidable hospitalizations within those counties. Future research should integrate data on cross-provincial medical expense settlements to more comprehensively characterize the patterns of spatial distribution in border regions.

## 3 Results

### 3.1 Study population

From 2022 to 2025, healthcare institutions in Ningxia documented 5,470,620 inpatients, of whom 617,320 received a primary diagnosis corresponding to one of the 14 diseases present in the Chinese ACSCs list. The baseline characteristics of the study population, along with the chi-square test results (Table 2), indicated significant differences in sociodemographic and clinical characteristics between the ACSCs and non-ACSCs groups (*p* < 0.001). Compared with the non-ACSCs group, the ACSCs group had a greater proportion of older adults (≥65 years), males, and rural residents, as well as a greater burden of comorbidities, as evidenced by a significantly greater proportion of individuals with a CCI score ≥1. In terms of health insurance, a greater percentage of patients in the ACSCs group were enrolled in UEBMI and Urban and Rural Resident Basic Medical Insurance, whereas the proportion of those paying out-of-pocket was lower. Collectively, these differences delineate a typical profile of ACSCs inpatients as “older adults, male, rural residents, with multiple comorbidities, and primarily covered by urban and rural resident basic medical insurance”.

**Table 2:** Contrasting multilevel traits in ambulatory care sensitive conditions (ACSCs) vs. non-ACSCs.

Variables	ACSCs-related hospitalizations *n* = 617,320 *n* (%)	Non-ACSCs-related hospitalizations *n* = 4,853,300 *n* (%)	*p* value
Sex			<0.001
Male	321,624 (52.1)	2,164,572 (44.6)	
Female	295,696 (47.9)	2,688,728 (55.4)	
Age			<0.001
0–15	25,114 (4.1)	662,457 (13.6)	
15–25	7,540 (1.2)	203,536 (4.2)	
25–45	55,095 (8.9)	929,323 (19.1)	
45–55	77,806 (12.6)	723,994 (14.9)	
55–65	121,015 (19.6)	878,602 (18.1)	
≥65	330,750 (53.6)	1,455,388 (30.0)	
Marital status			<0.001
Unmarried	41,360 (6.7)	868,741 (17.9)	
Married	519,166 (84.1)	3,727,334 (76.8)	
Widowed	40,126 (6.5)	140,746 (2.9)	
Divorced	7,408 (1.2)	48,533 (1.0)	
Others	9,260 (1.5)	67,946 (1.4)	
Occupation			<0.001
employee of government agency/enterprise/public institution	24,398 (4.0)	337,584 (7.0)	
Worker	13,298 (2.2)	134,834 (2.8)	
Farmer	268,786 (43.5)	1,548,045 (31.9)	
Self-employed/Freelancer	16,680 (2.7)	159,075 (3.3)	
Unemployed	76,811 (12.4)	675,670 (13.9)	
retiree	75,514 (12.2)	424,993 (8.8)	
Others	141,833 (23.0)	1,573,099 (32.4)	
Health insurance			<0.001
UEBMI	190,335 (30.8)	1,554,551 (32.0)	
URRBMI	367,398 (59.5)	2,533,374 (52.2)	
Out-of-pocket	29,956 (4.9)	544,485 (11.2)	
Other insurance	29,631 (4.8)	220,890 (4.6)	
CCI score			<0.001
0	197,168 (31.9)	3,137,080 (64.6)	
1–2	295,938 (47.9)	1,286,898 (26.5)	
3–4	107,224 (17.4)	274,725 (5.7)	
≥5	16,990 (2.8)	154,597 (3.2)	

This table presents data aggregated over the entire study period (2022–2025). ACSCs, ambulatory care sensitive condition; UEBMI, urban employee basic medical insurance; URRBMI, urban and rural resident basic medical insurance; CCI score, the Charlson Comorbidity Index score.

### 3.2 Rate of potentially avoidable hospitalizations and costs

The sex- and age-standardized annual average potentially avoidable hospitalization rate for inpatients in Ningxia from 2022 to 2025 (Table 3) was 505.37 per 100,000 population per year. Patients with chronic obstructive pulmonary disease (COPD) had the highest standardized potentially avoidable hospitalization rate (151.12 per 100,000 population per year), followed by those with diabetes (114.15 per 100,000 population per year) and chronic heart failure (54.09 per 100,000 population per year). Conversely, patients with anemia caused by iron deficiency had the lowest standardized potentially avoidable hospitalization rate, recorded at 1.50 per 100,000 population per year.

**Table 3:** Potentially avoidable hospitalizations rates and costs by ambulatory care sensitive condition.

Disease	Number of potentially avoidable hospitalizations	Standardized rate of potentially avoidable hospitalizations (per 100,000 population/year)	Average cost per hospitalization (CNY)	Total hospitalization cost (100 million CNY)
Chronic obstructive pulmonary disease (COPD)	189,873	151.12	4,749.48	10.47
Bronchial asthma	21,440	18.14	3,516.94	0.86
Hypertension	63,482	52.33	3,386.41	2.36
Chronic kidney disease	34,050	28.63	7,609.00	4.23
Diabetes	136,160	114.15	4,249.29	6.59
Bronchiectasis	12,600	10.43	4,458.35	0.72
Chronic heart failure	67,977	54.09	5,952.55	5.25
Atrial fibrillation	17,718	14.57	10,947.79	5.77
Chronic hepatitis B	6,714	5.80	3,340.60	0.26
Tuberculosis	13,808	11.31	7,467.01	1.28
Iron-deficiency anemia	1,778	1.50	4,176.89	0.08
Primary osteoporosis	10,089	8.06	6,105.17	0.88
Gastroenteritis	39,010	33.03	1,868.11	1.06
Influenza	2,621	2.22	2,143.80	0.10
sum	617,320	505.37	4,495.33	39.91

With respect to average hospitalization costs per admission, patients with atrial fibrillation incurred the highest average cost at 10,947.79 yuan, followed by those with chronic kidney disease at 7,609.00 yuan, those with tuberculosis at 7,467.01 yuan, and those with primary osteoporosis at 6,105.17 yuan. In contrast, patients with acute conditions, such as gastroenteritis (1,868.11 yuan) and influenza (2,143.80 yuan), experienced the lowest average hospitalization costs per admission. The per-case hospitalization cost for patients with diabetes was also relatively high at 4,249.29 yuan, reflecting the resource-intensive management required for patients with advanced stages of the disease. Among the core diseases, the per-case hospitalization cost for patients with hypertension was the lowest at 3,386.41 yuan.

From 2022 to 2025, the total direct medical expenditures for inpatient care related to ACSCs in Ningxia reached 3.991 billion yuan (approximately $554 million). The total inpatient costs and average cost per admission for each disease are presented in Table 3. Notably, patients hospitalized for COPD incurred the highest total inpatient costs, amounting to 1,047 million yuan, which represents 26.23% of the total inpatient costs for treating ACSCs. Diabetes (659 million yuan) and atrial fibrillation (577 million yuan) followed, ranking second and third, respectively. Collectively, these three conditions accounted for nearly 60% of the total inpatient costs associated with ACSCs.

### 3.3 Spatial autocorrelation analysis

Using the local indicators of spatial association (LISA) analysis method proposed by Anselin (1995) (37), we examined the spatial association of potentially avoidable hospitalization rates among patients in various counties and districts (Figure 1). The local spatial autocorrelation (*p* < 0.05) of ten counties and districts was significant and was classified into four categories: “High-High” (HH) hotspots, including Dawukou District and Jingyuan County, along with their surrounding areas, all of which exhibited elevated rates of potentially avoidable hospitalizations; “Low-Low” (LL) cold spots, comprising Hongsibao District, Qingtongxia City, and Zhongning County, as well as their neighboring regions, all of which reported relatively low rates of potentially avoidable hospitalizations; “Low-High” (LH) trough areas, where Pingluo County and Xiji County exhibited low rates of potentially avoidable hospitalizations, in contrast to the high rates in surrounding counties and districts; and “High-Low” (HL) isolated areas, including Xingqing District, Litong District, and Tongxin County, where the rates of potentially avoidable hospitalizations were high, whereas surrounding areas reported low rates. The local Moran’s I values for the remaining counties and districts did not reach statistical significance (*p* > 0.05).

**Figure 1:**
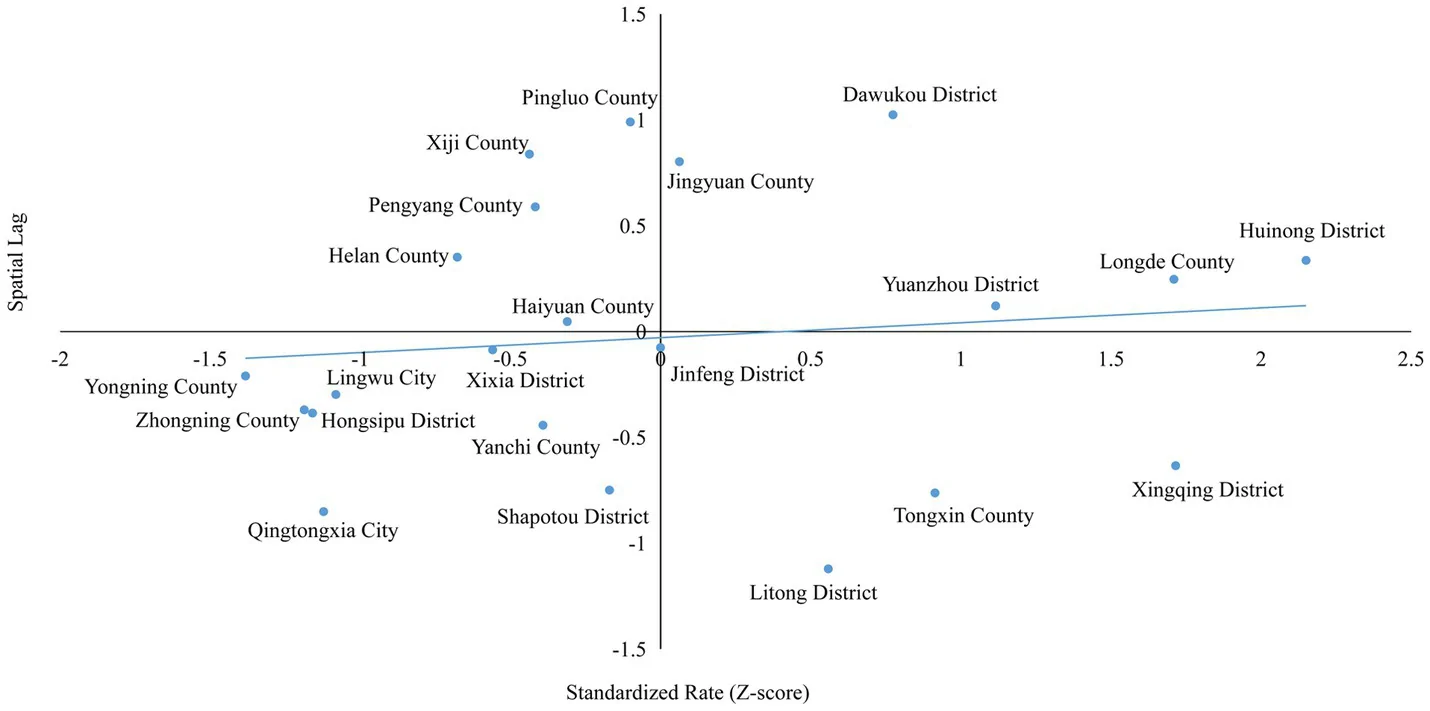
Moran scatter plot of potentially avoidable hospitalizations rates in Ningxia (2022–2025).

### 3.4 Analysis of factors influencing potentially avoidable hospitalizations

GEE, which incorporate both patient and county/district levels, were employed to identify factors influencing potentially avoidable hospitalizations among patients (Table 4). At the individual level, age exhibited a significant dose–response relationship with the risk of potentially avoidable hospitalizations. When the youngest age group (0–15 years) was used as the reference, the likelihood of potentially avoidable hospitalizations increased progressively with age. Patients aged ≥65 years had the highest likelihood of potentially avoidable hospitalizations (odds ratio [OR] = 4.367, 95% confidence interval [CI]: 4.248–4.489), suggesting that increased age is a significant risk factor. The CCI score reflects the severity of a patient’s comorbidities, exhibits a nonlinear inverted U-shaped relationship with the risk of potentially avoidable hospitalizations; patients with a CCI score of 3–4 had the highest likelihood of potentially avoidable hospitalizations (OR = 4.557, 95% CI: 4.505–4.610), whereas those with a CCI score of ≥5 exhibited a lower likelihood (OR = 1.589, 95% CI: 1.554–1.625). Compared with male patients, female patients presented a lower likelihood of potentially avoidable hospitalizations (OR = 0.786, 95% CI: 0.781–0.792). Compared with their unmarried counterparts, married patients (OR = 0.708, 95% CI: 0.695–0.721) exhibited a significantly lower likelihood of potentially avoidable hospitalizations. Patients enrolled in the Urban and Rural Resident Basic Medical Insurance (OR = 1.202; 95% CI: 1.191–1.212) were more likely to experience potentially avoidable hospitalizations compared with individuals enrolled in the UEBMI. With respect to occupation, workers, farmers, self-employed individuals/freelancers, and unemployed individuals exhibited a greater likelihood of potentially avoidable hospitalizations compared with employees of government agencies, enterprises, and public institutions.

**Table 4:** Results of the generalized estimating equations (GEE) for potentially avoidable hospitalizations.

Variables	OR	95% CI	*p* value
Sex (ref. male)			
Female	0.786	0.781–0.792	<0.001
Age, year (ref. 0–15)			
15–25	1.170	1.132–1.209	<0.001
25–45	2.009	1.954–2.065	<0.001
45–55	2.828	2.749–2.908	<0.001
55–65	3.209	3.121–3.300	<0.001
≥65	4.367	4.248–4.489	<0.001
Marital status (ref. unmarried)			
Married	0.708	0.695–0.721	<0.001
Widowed	0.951	0.930–0.972	<0.001
Divorced	0.862	0.835–0.890	<0.001
Others	0.759	0.737–0.781	<0.001
Occupation (ref. employee of government agency/enterprise/public institution)			
Worker	1.090	1.063–1.118	<0.001
Farmer	1.069	1.051–1.088	0.039
Self-employed/freelancer	1.193	1.166–1.222	<0.001
Unemployed	1.059	1.040–1.078	<0.001
Retired	1.001	0.983–1.021	0.888
Others	1.052	1.034–1.070	<0.001
Health insurance (ref. UEBMI)			
URRBMI	1.202	1.191–1.212	<0.001
Out-of-pocket	0.653	0.644–0.662	<0.001
Other insurance	1.111	1.091–1.131	<0.001
CCI score (ref. 0)			
1–2	2.672	2.651–2.694	<0.001
3–4	4.557	4.505–4.610	<0.001
≥5	1.589	1.554–1.625	<0.001
Log per capita GDP	0.846	0.705–0.947	0.038
PCP/1,000 pop	0.824	0.742–0.915	0.008
PC beds/1,000 pop	1.166	1.102–1.278	0.012

GDP, Gross Domestic Product; PCP/1,000 pop, number of primary care physicians per 1,000 population; PC beds/1,000 pop, number of primary care beds per 1,000 population. Results for log per capita GDP, PCP/1,000 pop and PC beds/1,000 pop were corrected using wild cluster bootstrap adjustment.

Analysis using the wild cluster bootstrap found that the direction of the effects of each variable was consistent with that of the original GEE model, and the associations with core macroeconomic variables remained stable (see Supplementary material). Compared with the traditional GEE model, bootstrap correction effectively mitigated the biases—such as underestimation of standard errors, excessively narrow confidence intervals, and false positives—caused by 22 small clusters. Statistical inferences were thus more conservative and reliable, providing further confirmation of the robustness and credibility of core conclusions of this study. A one-unit increase in the logarithm of per capita GDP at the county or district level was associated with an approximately 15% reduction in the likelihood of a patient experiencing potentially avoidable hospitalization (OR = 0.846, 95% CI 0.705–0.947, bootstrap *p* = 0.038). Additionally, for each additional primary care physician per 1,000 population, the likelihood of a patient experiencing potentially avoidable hospitalization decreased by 18% (OR = 0.824, 95% CI 0.742–0.915, bootstrap *p* = 0.008). For each additional primary care bed per 1,000 population, the likelihood of a patient experiencing potentially avoidable hospitalization increased by 16.6% (OR = 1.166, 95% CI 1.102–1.278, bootstrap *p* = 0.012).

## 4 Discussion

In this study, a China-specific list of ACSCs was applied for the first time to evaluate potentially avoidable hospitalizations in the resource-limited province of Ningxia, which is located in western China. This list was used to systematically assess the rates of potentially avoidable hospitalizations, direct medical costs, spatial distribution, and multilevel determinants associated with each disease category. The subsequent discussion focuses on four principal findings.

There are significant differences among countries in the lists of the ACSCs conditions used in studies, ICD coding rules, admission thresholds, healthcare systems, population demographics, and the denominators used to calculate rates; currently, there is no international consensus on a unified list of ACSCs (38). Therefore, the international reference data presented below are only provided for general background information and should not be considered strict benchmarks for comparison. This study found that the proportion of potentially avoidable hospitalizations among inpatients in Ningxia was approximately 11.28% (617,320/5,470,620 = 11.28%), a figure comparable to that of middle-income countries such as Malaysia and Brazil (39, 40). The standardized rates of potentially avoidable hospitalizations varied significantly across ACSCs, with the highest rate being 100 times greater than the lowest. These findings support the discriminant validity of China’s ACSCs list at the macro level. Among the different disease categories, patients with diabetes and COPD exhibited the highest rates of potentially avoidable hospitalizations, which is slightly lower than the OECD average (41, 42). However, a direct comparison of exact figures may be subject to bias due to differences in definitions and coding. The increased rates of potentially avoidable hospitalizations among patients with diabetes and COPD are closely linked to the natural progression of these diseases, which are marked by “long latency periods, asymptomatic early stages, and resource-intensive late stages”. These findings underscore the importance of early screening at the PHC level, via pulmonary function tests and blood glucose monitoring, as well as the necessity for long-term follow-up.

The “disease-specific concentration” of potentially avoidable hospitalization costs is a prevalent characteristic in the context of chronic diseases in both China and Ningxia. Between 2022 and 2025, patients with COPD, diabetes, and atrial fibrillation collectively represented nearly 60% of the total direct medical costs associated with potentially avoidable hospitalizations for ACSCs in Ningxia. This “disease-specific concentration” aligns closely with the findings of domestic studies (43–45). In 2018, approximately 70% of potentially avoidable hospitalization costs for chronic diseases in China were linked to COPD, diabetes, and hypertension (43). Data from 2020 indicated that the combined potentially avoidable hospitalization costs for COPD, angina pectoris, and diabetes accounted for 65.85% of the total costs (44). These findings suggest that targeted interventions for a limited number of disease categories can yield substantial health economic benefits. The rate of potentially avoidable hospitalizations for core conditions such as COPD and diabetes should be considered as an exploratory candidate indicator. The rate of potentially avoidable hospitalizations is influenced by multiple confounding factors, such as health status of the individual patient, comorbidities, and hospital admission criteria, and therefore cannot serve as a direct measure of failures in primary health care. By enhancing early screening at the PHC level, standardizing chronic disease management, and optimizing health insurance payment incentives, resources can be focused on conditions characterized by “high cost concentration and strong potential for intervention at the PHC level,” thereby achieving the dual objectives of cost containment and quality improvement (45).

LISA was used to identify several clustering patterns, which challenge the conventional approach of uniform, region-wide interventions. Based on the results of the exploratory analysis using LISA, we propose the following research hypotheses: The emergence of HH hotspots, specifically in Dawukou District and Jingyuan County, may be closely associated with socioeconomic factors. A study conducted in southwest Sydney, Australia, revealed that hotspots for potentially avoidable hospitalizations were significantly more socioeconomically disadvantaged and had markedly lower access to integrated health services compared with cold spots (46). Another study noted that socioeconomic disadvantage is one of the most significant factors explaining spatial clustering (47). This means that simply improving the physical accessibility of healthcare services and enhancing infrastructure—without addressing the deep-seated socioeconomic disparities in the region—will make it difficult to reduce the risk of potentially avoidable hospitalizations in high-demand areas at its root. However, the aforementioned mechanisms were not directly tested in this study. Qingtongxia City and Zhongning County (LL cold spots) are located in the northern Yellow River irrigation area where medical resources are relatively abundant; their lower potentially avoidable hospitalization rates may be related to a more adequate allocation of primary care physicians and more comprehensive medical facilities. The experiences of these “cold spots” may offer potential avenues for improvement in HH hotspots, but their transferability must be carefully evaluated in specific contexts. A nationwide registry study conducted in Finland indicated that regional disparities in potentially avoidable hospitalizations are not determined solely by primary care supply; even after controlling for supply-side factors such as primary care physician density, regional disparities persisted, suggesting that individual socioeconomic status, population health status, and the regional organization of hospital services also play significant roles (48). Therefore, when applying lessons from cold spots, it is necessary to further investigate the underlying multifaceted factors rather than simply replicating resource allocation models. Xingqing District and Litong District (the HL “island” region) are themselves regional centers within their respective cities, concentrating higher-quality medical resources. One possible interpretation is that the concentration of high-quality resources at regional medical centers may not automatically translate into a spillover effect on surrounding areas. However, this hypothesis requires further validation through patient flow data. Pingluo County and Xiji County (the LH “depression” region) have inherently low rates of potentially avoidable hospitalizations; regional medical centers in the surrounding areas may generate a siphon effect, attracting some patients to seek care across regional boundaries. However, this hypothesis requires verification using interprovincial medical settlement data. It should be emphasized that LISA analysis is an exploratory spatial data analysis method, and all of the above explanations should be regarded as research hypotheses rather than proven conclusions. Future research should integrate individual-level socioeconomic data, patient referral flow data, and more granular health resource distribution data, and it should employ methods such as spatial regression models or geographically weighted regression to directly test these hypotheses.

Among the individual-level factors influencing potentially avoidable hospitalizations in this study, advanced age and high CCI scores were identified as significant risk factors. Compared with men, women exhibited a lower risk of potentially avoidable hospitalizations, which aligns with findings from both domestic and international studies (49–51). Notably, patients with a CCI score of 3–4 exhibited the highest likelihood of potentially avoidable hospitalizations, whereas the likelihood decreased for those with a CCI score of ≥5, indicating an “inverted U-shaped” relationship. Previous explanations for this contradictory phenomenon have largely attributed it to coding bias or case-source bias, but this interpretation may not be comprehensive enough. From an epidemiological perspective, the competing risk mechanism may provide a more reasonable explanation; frail patients with extremely high complication rates are more likely to be hospitalized or die because of severe, nonambulatory care sensitive acute critical illnesses, which statistically and objectively lowers their odds of being hospitalized with an ACSCs as the chief complaint. Therefore, the likelihood of potentially avoidable hospitalizations is significantly reduced (52). This hypothesis could be validated in future studies using competing risk models such as the Fine–Gray model (52, 53). At the county and district levels, the number of primary care physicians per 1,000 population emerged as a protective factor against potentially avoidable hospitalizations. This finding underscores the substantially positive role of human resources in PHC institutions in preventing potentially avoidable hospitalizations, which is consistent with previous research findings (54, 55). At the systematic review level, van Loenen et al.’s analysis of 49 studies clearly revealed that an adequate supply of primary care physicians and improved continuity of care can significantly reduce potentially avoidable hospitalizations for conditions sensitive to chronic outpatient services (56). A nationwide study conducted in South Korea, which included 252 regions, revealed that a higher density of primary care physicians was associated with lower rates of hospitalization for ACSCs (57). Additionally, a longitudinal ecological study in Belo Horizonte, Brazil, indicated that a greater proportion of family medicine specialists correlated with reduced potentially avoidable hospitalizations rates; if all primary care physicians were family medicine specialists, the number of potentially avoidable hospitalizations would be projected to decline by 11.89% (58). In terms of domestic evidence from China, a study conducted in Sichuan Province revealed that for every 10% increase in the number of primary care physicians per 10,000 population, the standardized rate of potentially avoidable hospitalizations for diabetes decreased by 2.574 per 100,000 population (28). The protective effect of the number of physicians in PHC institutions on the prevention of potentially avoidable hospitalizations has been consistently validated across various countries and study designs. The number of primary care beds per 1,000 population has emerged as a risk factor against potentially avoidable hospitalizations, and international studies have consistently confirmed that an increase in hospital bed supply can drive demand for hospitalizations. A nationwide study in South Korea, a state-level systemic study in the U.S., and a cross-national analysis covering 23 countries revealed a positive correlation between hospital bed density and the rate of potentially avoidable hospitalizations (59, 60), and domestic nationwide data also indicated that hospitalization rates increase with increasing hospital bed supply (61). This relationship can be explained by Roemer’s Law: When the number of hospital beds is physically expanded without a corresponding increase in clinical competencies, the availability of beds itself may lead to unnecessary hospitalizations (62). In Ningxia, the proportion of primary care beds is low, and their utilization rate is not high; the increase in the number of primary care beds may reflect quantitative expansion rather than an increase in service capacity, as the corresponding human resources have not been strengthened in tandem. This finding contrasts with the protective effect of the number of primary care physicians observed in this study, suggesting that simply increasing the number of primary care beds without qualified physicians makes it difficult to fulfill the primary care gatekeeper role and may, in fact, increase the risk of potentially avoidable hospitalizations. Therefore, when allocating primary health care resources, limited resources should be prioritized for building the medical workforce and enhancing service capacity, rather than simply pursuing an expansion in the number of hospital beds. In this study, per capita GDP at the county and district levels was identified as a protective factor against potentially avoidable hospitalizations for patients. A comparative analysis of Italy and Germany revealed that the geographic distribution of hospitalization rates for chronic avoidable conditions (ACSCs) correlated with per capita GDP; during the study period, the risk of potentially avoidable hospitalizations for chronic ACSCs in Italy decreased by 23%, indicating that economic development and investment in PHC may have contributed to this trend (63). However, the relationship between economic development and potentially avoidable hospitalizations may exhibit complex characteristics across different populations. A study of older adults patients with hypertension in China reported that as per capita GDP increased, the number of medical visits increased, yet the likelihood of potentially avoidable hospitalizations also increased (29). This finding indicates that in certain populations, high economic levels may increase the detection rate of potentially avoidable hospitalizations by increasing access to medical care rather than by signifying a genuine decrease in risk. In resource-constrained areas of western China, the protective influence of per capita GDP may primarily operate through “increased public funding for PHC”.

### 4.1 Strengths and limitations

Strengths: First, this study represents the first real-world empirical application of the recently published China-specific list of ACSCs, directly addressing the research priorities identified by the authors of the original Delphi study (24). Second, hospital discharge records data from all healthcare institutions across the province were included, rather than relying on a sample or selected institutions; this mitigated selection bias and enhanced the generalizability of the study’s findings to the context of western China. Third, the four-year timeframe, coupled with detailed county-level spatial resolution, offers high-resolution evidence for the targeted allocation of resources.

Limitations: First, as an observational study, this research cannot establish a causal relationship between PHC factors and potentially avoidable hospitalizations. Second, the classification of potentially avoidable hospitalizations relies on administrative data rather than clinical chart reviews. Although the medical record system includes built-in checks to ensure consistency between clinical diagnoses and ICD-10 codes—thereby reducing human coding errors—a certain degree of misclassification bias may still exist. Third, the cost analysis encompasses only direct medical costs, specifically hospitalization expenses. Potentially avoidable hospitalizations incur significant indirect costs, including lost productivity, transportation expenses, and lost wages, which we could not quantify because of data limitations. Consequently, the true economic burden is likely much greater than our estimates. Fourth, the spatial analysis did not account for the boundary effects resulting from patients in certain counties and districts seeking medical treatment across provinces; this cross-provincial medical travel may lead to the misclassification of clustering patterns. Fifth, although the GEE method used in this study has relatively lenient requirements regarding the number of clusters and can partially mitigate estimation bias due to small-sample clustering through inference based on robust standard errors, its robust standard errors may still be conservative when the number of clusters is small. Consequently, while the effect estimates of county-level macro variables (e.g., per capita GDP and the number of primary care beds per 1,000 population) have been corroborated within the GEE framework, their precision remains limited by the adequacy of the available hierarchical structure information.

### 4.2 Policy implications and future directions

Policy implications: First, the ACSCs list could be explored as a supplementary technical tool for the analysis and evaluation of primary health care work, incorporated into the reference framework of relevant health policy research, and used to implement disease-specific and indicator-specific dual-precision management. It is recommended that potentially avoidable hospitalizations rates for the three core conditions—hypertension, COPD, and diabetes—be used as supplementary indicators for assessing county-level health status, providing local authorities with guidance on identifying potential areas for systemic optimization in chronic disease management. Second, differentiated strategies informed by the four spatial clustering patterns identified through LISA analysis should be employed. Spatially targeted interventions tailored to each county should be implemented, adhering to the principle of “one county, one policy.” For areas with high concentrations of HH, a comprehensive strategy that equally prioritizes “improving socioeconomic conditions” and “enhancing PHC service capacity” should be adopted. Third, health education and behavioral interventions for patients with diabetes, COPD, and hypertension should be enhanced to improve their self-management capabilities. Fourth, health insurance payment incentives linked to the performance of ACSCs should be investigated to facilitate the decentralization of service delivery. It is advisable to utilize the UK’s Quality and Outcomes Framework (QOF) (64) and, within the framework of capitation or performance-based payment reforms, connect the accomplishments of PHC institutions in reducing potentially avoidable hospitalization rates for hypertension, COPD, and diabetes to the retention of health insurance surpluses and performance-based rewards.

Future research: First, the cross-regional applicability of this checklist in eastern China and additional western provinces should be validated. Second, longitudinal analyses should be conducted to evaluate the dynamic effects of PHC policy interventions on potentially avoidable hospitalization rates. Third, behavioral targets in healthcare decision-making should be investigated by incorporating patient-level qualitative research. Fourth, cost effectiveness analyses of investments in human resources and equipment at the PHC level should be performed to inform optimal resource allocation. Fifth, data on cross-provincial medical expense settlements should be integrated and a spatial analysis model that accounts for boundary effects should be developed to more accurately characterize the geographic distribution of potentially avoidable hospitalizations in the future.

## 5 Conclusion

This study demonstrates the first application of the China-specific list of 14 ACSCs in the Ningxia region of western China, where a comprehensive real-world assessment was conducted to validate the feasibility and practical value of this list when applied to large-scale administrative data. The analysis revealed considerable heterogeneity in the hospitalization prevention rates across various diseases, with diabetes and COPD identified as the foremost priorities for enhancing PHC performance. Spatial analysis challenges the effectiveness of “one-size-fits-all” policies and advocates for localized, targeted interventions. A critical modifiable factor is the insufficient physician density in PHC institutions. These findings offer an empirical foundation for the dynamic updating of China’s ACSCs list and evidence-based resource allocation in resource-constrained regions of western China. However, when interpreting the findings of this study, researchers and policymakers should bear in mind that the rate of potentially avoidable hospitalizations is a population-level warning sign that reflects a complex interplay of factors involving patients, healthcare providers, the healthcare system, and socioeconomic conditions—rather than a direct measure of primary care quality.

## Data Availability

The raw data supporting the conclusions of this article will be made available by the authors, without undue reservation.
