# Supplementary material for: Assessment of potentially avoidable hospitalizations in Ningxia, China: application of a China-specific ACSCs list
Source: Front Public Health. 2026 Sep 29;14:1922200. doi: 10.3389/fpubh.2026.1922200 (PMC13658171; doi:10.3389/fpubh.2026.1922200)
Supplement: Supplementary file 1 [file Table_1.DOCX]

**Supplementary Material**

**Supplementary Table 1. Results of the wild cluster bootstrap for potentially avoidable hospitalizations**

| **Variables** | **OR** | **95% CI** | **P value** |
| --- | --- | --- | --- |
| **Sex (ref. Male)** |  |  |  |
| Female | 0.783 | 0.737–0.832 | <0.001 |
| **Age, year (ref. 0–15)** |  |  |  |
| 15–25 | 1.148 | 0.825–0.952 | 0.032 |
| 25–45 | 1.991 | 1.543–2.571 | <0.001 |
| 45–55 | 2.789 | 2.043–3.806 | <0.001 |
| 55–65 | 3.166 | 2.259–4.436 | <0.001 |
| ≥65 | 4.276 | 3.030–6.034 | <0.001 |
| **Marital status (ref. Unmarried)** |  |  |  |
| Married | 0.707 | 0.628–0.795 | <0.001 |
| Widowed | 0.951 | 0.826–1.095 | 0.482 |
| Divorced | 0.862 | 0.750–0.991 | 0.039 |
| Others | 0.764 | 0.775–0.902 | 0.042 |
| **Occupation(ref. employee of government agency / enterprise**  **/ public institution)** |  |  |  |
| Worker | 1.081 | 0.927–1.260 | <0.001 |
| Farmer | 1.066 | 1.047–1.075 | 0.042 |
| Self-employed/Freelancer | 1.179 | 1.034–1.345 | 0.014 |
| Unemployed | 1.041 | 1.013–1.065 | <0.001 |
| Retired | 1.016 | 0.875–1.181 | 0.833 |
| Others | 1.049 | 1.029–1.061 | <0.001 |
| **Health Insurance (ref. UEBMI)** |  |  |  |
| URRBMI | 1.210 | 1.012–1.446 | 0.036 |
| Out-of-pocket | 0.647 | 0.489–0.856 | 0.002 |
| Other insurance | 1.107 | 1.089–1.127 | <0.001 |
| **CCI score (ref. 0)** |  |  |  |
| 1–2 | 2.700 | 2.196–3.318 | <0.001 |
| 3–4 | 4.556 | 3.228–6.444 | <0.001 |
| ≥5 | 1.606 | 1.200–2.681 | <0.001 |
| Log per capita GDP | 0.839 | 0.831–0.846 | <0.001 |
| PCP/1,000 pop | 0.802 | 0.798–0.807 | <0.001 |
| PC beds/1,000 pop | 1.094 | 1.085–1.104 | <0.001 |

Note: GDP, Gross Domestic Product; PCP/1,000 pop, number of primary care physicians per 1,000 population; PC beds/1,000 pop, number of primary care beds per 1,000 population. Results for Log per capita GDP, PCP/1,000 pop and PC beds/1,000 pop were derived from generalized estimating equations (GEE).
